# Impact of flipped classroom model in increasing the achievement for medical students

**DOI:** 10.1186/s12909-023-04276-3

**Published:** 2023-04-27

**Authors:** Hanadi Abdelgadir Ahmed Sourg, Shahenaz Satti, Nasereldin Ahmed, Adil Ballal Mohammed Ahmed

**Affiliations:** 1 Faculty of Medicine, University of Khartoum, Khartoum, Sudan; 2 Faculty of Medicine, Al Neelain University, Khartoum, Sudan; 3 King Saud bin Abdulaziz University for Health Sciences, Riyadh, Kingdom of Saudi Arabia

**Keywords:** Flipped classroom model, Traditional lecture, Medical education, Technology

## Abstract

**Background:**

Over the past few years, there has been a parallel development in the use of the internet and technology for teaching purposes. The Flipped classroom model (FCM) used by the instructor aims at spending more time interacting with students rather than lecturing them. There are very few studies about the effectiveness of FCM on student performance and perception as compared to the traditional lecture in colleges of medicine. This study evaluates the effectiveness of the FCM on the academic achievement of students in terms of increased performance and perception as compared to the traditional lecture the medical students in Al-Neelain University-Sudan.

**Method:**

This case-control study compares using (FCM) in the medical students at Al-Neelain University and the traditional lecture and its effect on students’ academic achievement. The students were randomly assigned into two groups (A & B), flipped classroom group A (30 students as a test), and traditional classroom group B (33students as control). Major outcome indexes were pretest and posttest results used for students’ academic achievement performance assessment and a questionnaire used for student perception evaluation about the FCM. Finally, statistical analysis was performed using SPSS programs.

**Results:**

Although the pretest and posttest scores showed highly statistically differences within each group (A&B) with *P*<.000, when comparing the pretest and posttest scores of the studied groups showed that, there were no statistically significant differences between the pretest and posttest scores between them with *P*=0.912 and 0.100 respectively. However, more than 80% of participants were satisfied with using a flipped classroom. While more than 90% of students were more motivated to learn in flipped classrooms meeting learning targets when they used FCM.

**Conclusion:**

There was a positive student perception towards using the FCM, despite no significant effect of FCM on medical students’ academic achievement.

## Background

During the past recent years, education sector researchers have shown great interest and attention to the flipped classroom approach [1]. The FCM has been defined by researchers to have the core idea of focusing on students’ home study and saving time for class activities. Hamdan *et al* defined the flipped classroom as “shifting direct learning out of the large group learning space and moving it into the individual learning space, with the help of one of several technologies” [2].

Recently, there has been a shift in the teaching methodology in medical schools from the traditional approach to other educational approaches that encourage thinking and active participation, and contribution from students [3]. The flipped classroom has become a wide world approach since 2012 up to now. This approach focuses on student center learning rather than teacher center learning by using different types of technology outside the classroom and with more students- teachers interact inside the classroom [4]. Despite the many types of blending learning approaches, evidence suggests that FCM is a significant blending approach in medical professions education as it causes apparent improvement in students learning and performance versus the traditional learning methods [5].

The main aim of using the FCM is to engage students in active learning experiences through discussion and group communication and therefore use class time more efficiently [6]. Flipped classroom in the medical education field acts as a perfect tool and is suitable for students so that they contribute more actively and concentrate on class collaboration while using the pre-class time to get a large volume of information in their free time [7, 8].

In 2007, Bergmann and Sams were looking for a way to provide lectures for their students who missed classes. They achieved it by recording lectures and giving the records to the students. The method took developing processes to be the current method of flip learning in different educational subjects [9]. In 2011 about 16,000 worldwide professional participants who were interested in the flipped model education had a shared network. This created great support for the use of technology and its implementation in the education processes [10, 11]. Up till now, flipped model education has reached a high level due to continuous technological development.

Bloom's revised taxonomy theory was the basis of the FCM study. This theory has six levels of learning and is arranged from the lowest level below to the highest level at the top. Remembering, Understanding, Applying, Analyzing, Evaluating, and Creating. Through these levels the student tries to recognize and recall the information, understand the basic concepts, interpret the information and summarize what they have learned, apply knowledge to the actual situation, produce creative thinking and produce something new from what they have learned. Based on Bloom's revised taxonomy [12], the FCM had two components pre-class learning and in-class learning.

By applying FCM many benefits appear in education process development, especially at the higher education level. Baker reported many benefits for teachers when applying a flipped classroom. In summary, class time saving for class activities, more focus on students' understanding and application rather than recall of facts, students controlling their learning, giving students their learning responsibility, and student-to-student knowledge and information sharing [13]. Moreover, in 2012, Bergmann and Sams also listed several benefits include: assisting busy and stressed students, increasing teacher-student and student-student interaction, being friendly to students with diverse abilities, and enabling customizable and flexible instruction as well as inspiring, encouraging, listening, and provide vision to them [9].

Although FCM has an effective impact on students learning performance, engagement, and motivation as appeared in previous studies [14, 15], some studies showed the negative effect of FCM. In 2014, Kim *et al*. concluded that the student’s grade was not affected by FCM when it was used as a learning approach [16]. In 2016, Sun and Wu's study also showed no impact on interaction and learning satisfaction between students and instructors when FCM was used [17]. Similarly, in 2016, Smallhorn did not observe any variation in the academic achievement of a student with the use of FCM [18].

In per-clinical educational programs, the effectiveness of an FCR for teaching has not been rigorously investigated. and, to the best of our knowledge, no previous studies on the efficacy of FCR in Sudan have been conducted. In this study, we aimed to evaluate the effectiveness of the flipped classroom model on the students' achievement (performance and attitudes) of 2^nd^-year medical students at the Faculty of Medicine, Al-Neelain University as compared to the traditional lecture model.

## Methods

This quasi-experimental study was conducted in the Faculty of Medicine, Al-Neelain University Khartoum, Sudan in November 2019. sixty-three 2^nd^ year, medical students were recruited.

Thirty students were randomly allocated to experimental group A (12 females and 18 male students) and 33 students to control group B (18 females and 15 male students). The topic of the lecture was asthma, which was a new topic for all students at this level. Inclusion criteria: Second-year students, Faculty of Medicine, Al-Neelain University. While any student who did not attend the class and any student of the experimental group who did not read the learning material were all excluded from the studied groups.

### Pretest

All students (A and B) took 10 min pre-test before class started.

### Group A

The topic-specific objectives, learning materials, five-minute video, and 20-minute, slides as a summary of the topic, were given to group A two days before the class the student should go through all reading materials.

In class, the session started with questions given to each student individually, and they receive their score without knowing which questions are correct or incorrect individual readiness awareness test (iRAT) . Students were then divided into groups to take the same test and students are allowed to debate the issues and teach concepts to one another (team readiness, awareness test (tRAT), then a discussion using the PowerPoint template (PPT) and conclusion. Application exercises done by each student write what he /she understands from the lecture and reading materials (concept recall) and each student writes a question from the lecture and passes it to his/her neighbor.The iRAT and tRAT were conductued in Socrative which is an on line application in which you can conduct quizzes, surveys, team activities, and educational content also can be uploaded in to the application so student can have an access to it prior to scheduled session.

### Group B

The second group (B) received the formal lecture (PPT) for fifty minutes, followed by a ten minutes discussion, then the group was provided with the same learning material given to group A.

### Posttest

The two groups were given the same pretest questions, one day later to compare and measure the achievement of both groups.

The two tests were designed to estimate the number of relevant concepts that students can recall at the end of the lecture (i.e. questions were related to lecture objectives). The students were expected to form links of concepts correlated to the different learning items and relate the new concepts to their prior knowledge. Any differences that appear in the test results should be the result of the experimental variable for student performance assessment.

### Subjects questionnaire

A questionnaire was given only to group (A) students who were taught in the flipped model to assess their perception of the FCM using google form. https://docs.google.com/forms/d/e/1FAIpQLSfMEXOevUYDazok7NzIkbquRz_sUEm9GGGhbSZ9O00DAuM_g/viewform?usp=sf_link).

The learning materials were given to group A before the lecture while group B attended a traditional lecture as planned. The pretest and posttest were the same tests which were a concept recall test of 10 multiple-choice questions (MCQs). Any student was given full marks if he/she mentioned all the points.

In this study, the statistical evaluation was performed using SPSS (version 20, Chicago, SPSS Inc. USA). The descriptive statistics for the numerical variables were presented as means and standard deviation after being assessed for normally distributed variables which were examined using the Shapiro-Wilk test. The t-test analysis was used to determine the significant difference that exists between the pretest and posttest scores of each student group. Then, an independent t-test was used to determine the significant difference that exists between the traditional and flipped classroom pretest and posttest score means. *P* < 0.05 was considered significant. The student perception questionnaire was used to evaluate the flipped classroom method. Likert scale was used to gather feedback and each question was a 5-point Likert item from “strongly disagreed” to “strongly agreed”. Cronbach^’^s alpha was used to measure the questionnaire reliability which was 0.845 which indicated a high level of internal consistency. Responses to the Likert-type questions were analyzed with descriptive statistics.

The ethical clearance of this study was approved by the Ethical Review Committee - Al-Neelain University Board, Khartoum, Sudan. All the studied volunteers enrolled in this study signed informed consent, and confidentiality and privacy of data were considered.

## Results

Of the 63 participants, females’ percentages were 60 and 54.5% in group A and group B respectively (Table 1). Although the pretest and post-test scores showed statistical differences within each group (Tables 2 and 3). The mean ±SD of pretest scores of group A and group B was 45. 33±17. 167 and 44.85±17.522, respectively. While the mean ±SD of post-test scores of A group and group B were 78.00±15.177 and 71.82±14.242, respectively (Table 4). The independent t-test which compared pretest and post-test scores of the studied groups showed that, there were no statistically significant differences between the pretest and posttest scores between group A and group B with *P* = 0.912 and 0.100 respectively (Table 5).

**Table 1:** Distribution of the gender among the two groups

Gender	Flipped Classroom lecture group A			Traditional lecture group B		
	Frequency	Percentage	Total	Frequency	Percentage	Total
Female	12	60	30	18	54.5	33
Male	18	40	100	15	45.5	100

**Table 2:** The comparison between pretest score and posttest score of group A

			Pretest Score – Posttest Score
Paired Differences	Mean		-32.667
	Std. Deviation		17.798
	Std. Error Mean		3.250
	95% Confidence Interval of the Difference	Lower	-39.313
		Upper	-26.021
T			-10.053
Df			29
Sig. (2-tailed)			.000

The paired t-test showed significant differences between the pretest and posttest scores of group A with t [29] = -10.053, *P*<.000

**Table 3:** The comparison between pretest score and posttest score of group B

			Pretest Score – Posttest Score
Paired Differences	Mean		-26.970
	Std. Deviation		18.283
	Std. Error Mean		3.183
	95% Confidence Interval of the Difference	Lower	-33.453
		Upper	-20.487
T			-8.474
Df			32
Sig. (2-tailed)			.000

*P*<0.05

**Table 4:** The mean ±SD of pretest scores and posttest of the studied groups

**Group Statistics**					
	Subject	N	Mean	Std. Deviation	Std. Error Mean
Pretest Score	Flipped Classroom lecture group A	30	45.33	17.167	3.134
	Traditional lecture group B	33	44.85	17.522	3.050
Posttest Score	Flipped Classroom lecture group A	30	78.00	15.177	2.771
	Traditional lecture group B	33	71.82	14.242	2.479

**Table 5:** Comparison of pretest and posttest scores of then studied groups

			Pretest Score		Pottest Score	
			Equal variances assumed	Equal variances not assumed	Equal variances assumed	Equal variances not assumed
Levene’s Test for Equality of Variances	F		.039		.248	
	Sig.		.844		.620	
t-test for Equality of Means	T		.111	.111	1.668	1.663
	Df		61	60.645	61	59.475
	Sig. (2-tailed)		.912	.912	.100	.102
	Mean Difference		.485	.485	6.182	6.182
	Std. Error Difference		4.378	4.373	3.707	3.718
	95% Confidence Interval of the Difference	Lower	-8.269	-8.261	-1.230	-1.257
		Upper	9.231	9.231	13.594	13.621

All thirty flipped classroom group A students responded to the questionnaire. The responses to each statement are summarized in (Table 6). No one of the participants strongly disagrees or even disagreed about the use of FCM but more than 80% of participants were satisfied with using flipped classrooms (Agreed: 63.3% and Strongly agreed: 20.0%).

**Table 6:** Frequency and percentage of students’ responses to questionnaire questions

	Strongly Disagreed		Disagreed		Neutral		Agreed		Strongly Agreed	
	^a^Fre	^a^Perc	Freq	Perc	Freq	Perc	Freq	Perc	Freq	Perc
Q1	0	0	0	0	7	23.3	9	30.0	14	46.7
Q2	0	0	1	3.3	4	13.3	14	46.7	11	36.7
Q3	0	0	3	10.0	7	23.3	9	30.0	11	36.7
Q4	3	10.0	8	26.7	9	30.0	6	20.0	4	13.3
Q5	0	0	1	3.3	11	36.7	12	40.0	6	20.0
Q6	0	0	1	3.3	8	26.7	12	40.0	9	30.0
Q7	3	10.0	11	36.7	7	23.3	5	16.7	4	13.3
Q8	0	0	1	3.3	1	3.3	23	76.7	5	16.7
Q9	0	0	0	0	5	16.7	19	36.3	6	20.0
Q10	0	0	1	3.3	7	23.3	12	40.0	10	33.3
Q11	0	0	3	10.0	7	23.3	13	43.4	7	23.3
Q12	0	0	1	3.3	4	13.3	19	63.3	6	20.0
Q13	0	0	3	10.0	3	10.0	14	46.7	10	33.3
Q14	0	0	1	3.3	7	23.3	15	50.0	7	23.3
Q15	0	0	1	3.3	8	26.7	15	50.0	6	20.0
Q16	7	23.3	10	33.3	7	23.3	2	6.7	4	13.3
Q17	4	13.3	5	16.7	10	33.3	8	26.7	3	10.0
Q18	2	6.7	1	3.3	5	16.7	14	46.7	8	26.7

Rating Scale: 5. Strongly Agreed, 4. Agreed, 3. Neutral, 2. Disagreed, 1. Strongly Disagreed

^a^*Fre* Frequency, *Perc* Percentage

The majority of students perceived that FCM gives them greater opportunities to do activities in class to the best of their abilities. Therefore, they completed their application exercises to the best of their ability (Strongly agreed: 46.7%) as well as completed and collaborated in the discussion (Agreed: 40.0% and Agreed: 43.3% respectively). When comparing students’ activities done easier in FCM or when doing it with traditional lectures like application of exercises and discussion, about 80% or more answered that agreed: 46.7% and strongly agreed: 36.7% when they used FCM in application exercises as well as in discussion (Strongly agreed: 36.7%). About 50% or more of the students' performance and attitude were affected by FCM and appeared immediately when students applied exercise and collaborated in the discussion (Agreed: 63.3% (performance) and Agreed:50.0% (attitude) respectively). Not only were students affected positively by FCM, but around 40% of students became better at performance and attitude (Agreed: 40.0% of performance and Agreed: 40.0%for attitude). More than 70% of the students agreed that their performance and attitude rate increased by using FCM.

However, the presence of stress (Disagreed: 26.7%) and discomfort (Disagreed:36.7%) decreased when students collaborated in discussion or tried to complete the application exercises when they used FCM. Around 30% of students only (strongly agreed: 10.0% and agreed: 26.7) need help from the instructor to understand lectures by flipped classroom module more than by traditional lectures. However, about 20% only of students thought that technology made flipped classroom use more difficult (strongly agreed: 13.3% and Agreed:6.7%). Finally, more than 90% of students were more motivated to learn in flipped classrooms and meet learning targets when they used FCM.

## Discussion

The main aim of the present study was to evaluate the effectiveness of the flipped classroom model (FCM) on achievement in terms of increased performance and attitudes of students as compared to the traditional lecture.

### Students' academic achievements

Our findings showed that the pretest and posttest scores showed highly statistically differences within each group separately, but the independent t-test which compared the pretest and post-test scores of the studied groups revealed that there were no statistically significant differences between group A (the FCM group) and group B (Traditional Lecture group) pretest and posttest scores. This may be explained by the fact that the Traditional Lecture group was given reading materials to study immediately after the lecture and before the post-test, which we think is biased toward the method since no reading materials are usually given after lectures. In other words, the Traditional Lecture group studied the reading materials and was immediately given the post-test, which is not the real practice during the traditional lecture. Another explanation for the absence of difference between the two groups may be that FCM was a new experience for the FCM group hence they were facing this new approach for the first time in their education and the time used was very short for students' preparation.

This explanation goes with Chen Hsieh et al study as they mentioned that, the students facing difficulty dealing with the FCM because it was a new approach to them, and most of the students stated that using FCM needed heavily loaded requirements besides they had no time to watch videos outside the class [19]. The results of the present study were showing similar results as those shown in other studies [20, 21] that, no significant differences between FCM and traditional models were found in students’ academic achievement [22].

However, some studies showed that the use of FCM increased students' academic success. Janotha in 2016 was examining if the FCM teaching approach affects the academic achievement of nursing students. Students were administered a national standardized test and Council of Health Education System tests. The result of the study showed that the students in the experimental group achieved higher academic performance than the students in the control group (Janotha, 2016) [23]. Similarly, another study was conducted by Zengin in 2017, to determine the effect of the flipped classroom approach designed by using Khan Academy and free open-source software on students’ academic achievement and to examine students’ views about this approach [15]. The learning environment was designed using the FC Model alongside Khan Academy and free open-source software. Twenty-eight students participated in the Mathematics Teaching Program at the stated university and the results showed that there was double the students’ academic success besides that the new approach facilitated student learning and enabled visualization in mathematics teaching. While in 2012 Pierce concluded that, some of the factors that may contribute to students’ improved scores like student mediated contact with the course material before classes and the interactive class activities. So, the implementation of the FCM to teach a renal pharmacotherapy module resulted in improved student performance and positive student perceptions towards the flipped classroom approach [24]. Another study in the introductory physiology class done by Entezari and Javdan (2016), found that performed on exams better by community college students when used FCM than students in a traditional model taught the same course [25].

On the other hand, some researchers used a combination of different methods like problem-based learning (PBL) with FCM and the result was a positive student academic achievement. Like Xiaolei et al. study in 2019, they combined PBL with FCM in a hyperthyroidism endocrinology internship at Medical College and concluded that this combination although it increased students’ workload, it was better than using traditional lecture alone [26]. Moreover, Tune *et al* concluded in their study that FCM is more effective in medical students [27]. Further, the Chun et. al. study also used a combination between flipped classroom approach and team-based lecture- and evidence-based learning for ophthalmology teaching for eight-year program students concluded that no significant difference was found in the final theoretical test scores for both groups, but this combination of significantly improved the students' performance. Also in the same study, teachers and students approved together to use of this combination and commented that the course helped them to develop skills in creative thinking, problem-solving, and teamwork [28].

So, to do the successful implementation of FCM and get better student performance three suggested points by Ramula may be necessary: the first teacher should select suitable topics, time match the optimum of students’ capability, and have well-planned classroom discussion [29]. Khe and Chung suggested that the flipped classroom approach yields significant improvement in student learning compared with traditional teaching methods when used in different disciplines of health professions education [30]. Humaira conducted a study on first-year MBBS students and stated that there were many goals achieved by using FCM proper steps like promoting a problem-solving approach, deep understanding of concepts, and active interaction between students and instructors. The author also thought that the FC had popularity in medical education, but still, the published evidence is deficient in FCM effectiveness in academic achievements [31].

The effectiveness of FCM is still controversial and the different subjects or course designs might be the causes of the heterogeneity among these studies [32–34].

However, many researchers showed improvement significantly in learning when they used FCM, and the learning outcome of FCM by many previous reviews suggested that improvement in student performance was found when compared with the traditional teaching approach [35, 36]. Findlay et. al. explained the better learning experience and acceptance by students to the flipped classroom may be due to that students felt they had more opportunities in class to ask questions to the teacher or their class colleagues. Moreover, to get a more effective flipped classroom several processes were suggested by Findlay et. al. study which summarized the points: the purpose of the flipped classroom approach must be understood first by the students, the students' opportunity and their responsibilities to this new style of learning should express by teachers to students, instructors should be well trained and students should be committed to new learning method [37].

### Student perceptions towards the flipped classroom approach

In this study, the students generally strongly agreed and agreed with using the flipped classroom approach, and more than 80% of the group (A) students were satisfied with using flipped classroom because they had participated well in-class activities, feel more motivated, and had good interaction with classmate and teacher. Moreover, the students’ qualitative comments that the majority of students perceived that FCM gives them greater opportunities to do activities in class to the best of their abilities. Further, no one of the participants strongly disagrees or even disagreed about the use of FCM. This result agrees with Morgan et al hence most of their study subjects consider FCM as an effective learning tool and are satisfied by using it [38].

However, Zhao et al reported that about half of the participants preferred to use FCM while another half did not [39]. The latter result is as similar to S K Gubbiyyapa et al and Veeramani et al studies [40, 41]. Moreover, Missildine et al found that, there was a more positive perception of students towards FCM using but that not necessarily to be satisfied by using it [42].

In the present study, the comments given by the students can be considered because these comments reflect their acceptance of this flipped class method. Most of the students commented positively since they participated well in-class activities, feel more motivated, and had good interaction with classmates and teachers. Moreover, greater opportunities to do activities in the class were available to the students when using FCM. Therefore, about half of the students completed their application exercises to the best of their ability as well as completed and collaborated in the discussion with their colleagues. That result agrees with an Indian study that used FCM in anatomy topics and reported that 80 % of students' perception was agreeing to use FCM because they had an opportunity to interact and communicate with their instructors and peers in class and be more engaged by this new method. Also in the same study, the use of Audiovisual help in an effective learning process, and more than 50% of medical students were strongly agreed and interested in using lecture videos as we used in this study [43].

In summary, the results of this study indicate that majority of the students favored the flipped classroom approach over the traditional classroom approach. Besides, the flipped classroom approach was more effective than the traditional classroom in increasing student learning performance and attitude despite the negative impact of the flipped classroom approach on students’ academic achievement.

### Study limitations

The present study did not investigate the relevant issues with a larger sample size and longer-time experiments in Sudan or other countries.

## Conclusions

This study aimed to evaluate the impact of the flipped classroom method on the subjects compared with traditional teaching methods from two different views: one the impact of FCM on students’ academic achievement and other impact on the students’ performance and attitude perception. After analyzing the results, some conclusions can be raised:

There were no statistically significant differences between the pretest and posttest scores between the FCM group and the traditional model group. The explanation for that, maybe due to the FCM was a new experience for group A (FCM group) hence they are facing this new approach for the first time in their education, and time was very short for preparation. However, the students’ opinion towards the FCM was positive and they were satisfied with the model because they had participated well in-class activities, feel more motivated, and had good interaction with classmates and the teacher. Furtherly, the majority of the students’ performance and attitude rates increased by using FCM. Moreover, not only were students affected by FCM only but also became better in performance and attitude because they had an opportunity to interact and communicate with their instructors and peers in class and be more engaged by this new method. Finally, more than 90% of students were more interested to learn in flipped classrooms and meeting learning targets. So, without consideration of the test scores, FCM is maybe better than the traditional one. i.e. in short, the FCM appears more on students learning activities than on the grading tasks . Future research should concentrate on the challenges that the FCM faces and the best way to overcome these challenges.

## Data Availability

The data supporting the present findings are contained within the manuscript.

## Abbreviations

FCM: Flipped classroom model
IRAT: Individual readiness awareness test
MCQs: Multiple choice questions
PBL: Problem based learning
PPT: Powerpoint templates
TRAT: Team readiness awareness test
SD: standard deviation
SPSS: Statistical package program for social science
